# Clinical Practice Recommendations for Non-Dermatologists on the Diagnostic Suspicion of GPP

**DOI:** 10.3390/jcm15156087

**Published:** 2026-08-05

**Authors:** Antonella Di Cesare, Elia Rosi, Annalisa Cavallo, Serena Guiducci, Anna Lucia Marigliano, Simone Vanni, Francesca Prignano

**Affiliations:** 1 Dermatology Unit, USL Toscana Centro, Ospedale P. Palagi, Viale Michelangelo, 41, 50125 Florence, Italy; 2 Section of Dermatology, Department of Health Sciences, University of Florence, University of Florence Ospedale P. Palagi, Viale Michelangelo, 41, 50125 Florence, Italy; 3 Infectious and Tropical Diseases Unit, AOU Azienda Ospedaliero Universitaria Careggi, 50134 Florence, Italy; 4 Department of Experimental and Clinical Medicine, Section of Rheumatology, AOU Careggi, University of Florence, 50134 Florence, Italy; 5 Pharmaceutical Governance and Appropriateness of Prescription Unit, Drug Department, USL Toscana Centro, 59100 Prato, Italy; 6 Department of Experimental and Clinical Medicine, High Dependency Unit, Careggi University Hospital, University of Florence, 50134 Florence, Italy

**Keywords:** GPP, flares, psoriasis, emergency, spesolimab

## Abstract

Generalized pustular psoriasis (GPP) is a rare, potentially life-threatening, chronic cutaneous inflammatory disease characterized by unpredictable, recurrent acute flares of painful sterile pustules on a widespread erythematous background. In addition to cutaneous manifestations, patients may experience fever, pruritus, pain, chills, and general malaise, which may be further complicated by secondary infection, sepsis, and organ failure, thus requiring urgent medical treatment and, in some cases, hospitalization. Prompt therapeutic management of the acute phase is crucial for severe cases, and proactive treatment to prevent flares should always be considered. However, early recognition of acute flares can be challenging due to the low frequency of the disease, the rapid onset of flares, the lack of hematological biomarkers and the absence of standardized diagnostic criteria. Moreover, despite the approval of new targeted therapies, there are still several unmet needs, as these treatments are highly expensive, not always readily available, and may have limited efficacy in patients with advanced or complicated disease. For these reasons, multidisciplinary round-table discussions and shared diagnostic and therapeutic algorithms involving dermatologists, who are responsible for diagnosing and treating GPP, and other medical specialists are desirable to facilitate prompt referral to dermatologists for accurate diagnosis and appropriate treatment. We report the updated literature discussed during a multidisciplinary meeting with the aim of providing practice recommendations for clinicians involved in GPP management.

## 1. Introduction

Generalized pustular psoriasis (GPP) is a rare and severe manifestation of psoriasis, typically characterized by the presence of sterile and painful sub-corneal pustules that can coalesce into lakes of pus. GPP can manifest in the presence or absence of plaque psoriasis and/or cutaneous or general systemic inflammatory symptoms. The clinical course of GPP is heterogeneous, and often unpredictable, with acute, relapsing or persistent flares [1,2,3]. The degree of skin involvement, the rapidity of pustule spreading, the intensity of inflammation, and the presence of systemic symptoms such as fever, general malaise, blood cell abnormalities and internal organ involvement are key drivers in patients’ prognosis and overall survival [1,4].

Hence, early recognition and treatment of GPP and of its flares are essential for dermatologists and physicians involved in GPP management. Raising awareness of the disease, from its epidemiological prevalence in the general and in the psoriatic population, to the triggers, frequency and clinical patterns of flares is paramount. Furthermore, improving the accuracy of diagnosis of both cutaneous and systemic manifestations, as well as optimizing therapeutic strategies, should be an upcoming goal in order to reduce the risk of complications, such as sepsis or multi-organ failure and their potentially life-threatening consequences [4].

Recently, the molecular mechanisms underlying GPP have been better investigated, identifying IL-36 signaling as the pivotal pathway in GPP pathogenesis [5,6,7]. These findings suggest that GPP has a distinct and specific pathogenesis compared with the well-established IL-23/Th17 signaling axis, dominant in plaque psoriasis. These discoveries led to the development and therapeutic approval of the first specific biologic agent (i.e., spesolimab) [8], while others are under investigation, offering novel opportunities to achieve both rapid and sustained disease control and to reduce the severity and frequency of flares [9,10,11].

Overall, GPP has a high socioeconomic burden not only from patients’ perspectives, due to profoundly impaired quality of life and psychological distress, but also from a healthcare point of view. This impact is even more substantial when considering healthcare resource utilization, the need for hospitalization, the number of visits, laboratory examinations, and direct and indirect treatment costs [12,13,14,15].

To address these clinical gaps and to support a more individualized approach to patient care, we report relevant data on current knowledge and practical recommendations on GPP. These insights were developed through a multidisciplinary meeting bridging together specialists with expertise in GPP, with the aim of providing practical, easy-to-use diagnostic and therapeutic guidance for routine clinical practice.

## 2. Methods

As a first step, three expert dermatologists performed a comprehensive and systematic literature search on the PubMed database.

The primary objective was to identify peer-reviewed articles published up to the face-to-face meeting held in November 2024. The search strategy focused on “generalized pustular psoriasis” or “GPP” as core search terms, combined with specific keywords including “epidemiology”, “diagnosis”, “differential diagnosis”, “management”, “emergency”, “flare”, “treatment”, “guidelines”, “consensus”, “comorbidities”, “arthritis” and “triggers”. No search restrictions were applied. To warrant the inclusion of the most recent scientific advancement, a further literature update covering publications up to April 2026 was performed and discussed as well.

To ensure high scientific rigor of our search, only relevant articles, published in English and involving human subjects, were selected. Articles were selected among the following categories: clinical trials, registry or database analyses, observational studies, literature reviews, consensus studies, case studies, case reports, and case series. Editorials, narrative reviews and commentaries were excluded.

After a preliminary abstract screening, a full-text screening was performed by two independent reviewers to exclude articles that were not relevant or did not meet the predefined inclusion criteria. In case of disagreement, the final decision was discussed during the meeting, with a third reviewer leading the selection.

Data extracted included epidemiology, pathogenesis, clinical features, triggering factors, comorbidities, differential diagnosis, laboratory tests, prognosis and treatment of GPP.

The collected papers were thoroughly summarized and presented during two meetings: a preliminary in-person round-table and a final virtual meeting. During these meetings, a multidisciplinary panel consisting of three dermatologists, a rheumatologist, an infectious disease specialist, an emergency medicine doctor and a hospital pharmacist reviewed and discussed both the updated literature and their clinical experience on GPP. This expert panel ensured that the topic was examined from different clinical perspectives. The main results and conclusions from these meetings, together with a summary of the relevant literature, were used to develop clinical practice recommendations for the early recognition and prompt intervention on GPP presented in this article.

## 3. Epidemiology of GPP

The epidemiological landscape of GPP is highly variable and complex, largely because of geographic differences, diverse genetic backgrounds, and the historical absence of specific registries all over the world. Furthermore, the lack of standardized criteria often hampers the proper clinical differential diagnosis between GPP, chronic plaque psoriasis, and other non-psoriatic pustular diseases, leading to potential underreporting or misclassification.

Studies conducted across European countries have consistently reported a notable increase in GPP prevalence over time. In the UK, the annual prevalence of GPP increased from 1.61 cases/million to 3.00 cases/million inhabitants from 2008 to 2019 [16], while a recent epidemiologic study in France found that GPP affected up to 52 cases/million in 2018, compared with an estimated 1.76 cases/million inhabitants estimated in 2004 [17].Recent data collected from other European countries, including Italy, Spain, Sweden and Germany reported a GPP prevalence of 1-to-3, 13.05, 15.3 and 50-to-100/cases/million inhabitants respectively, highlighting considerable heterogeneity across the continent. The establishment of national registries will be essential to obtain more reliable prevalence estimates [13,18,19,20].

In American countries marked differences in GPP frequency have been reported, ranging from 0.7 to 0.89 cases/100.000 inhabitants in Brazil [21] up to 150 cases/million inhabitants in the United States [22]. A broad variability is also observed in Asian-Pacific countries, where the reported prevalence ranges from 7.2 to 198 cases/million inhabitants in Japan and Malaysia respectively [23,24,25]. This variability may reflect differences in diagnostic practices and regional genetic predispositions.

Regarding its association with other forms of psoriasis, GPP has a prevalence ranging from 0.6 to 2.4% among patients with pre-existing plaque psoriasis [26]. Concurrently, up to 85.5% of GPP patients present a personal history of psoriasis that usually preceded GPP onset [13,27,28,29,30,31].

Recent advances in the understanding of GPP etiopathogenesis, together with the development of disease-specific therapies, have significantly increased clinical attention toward the disease. Accordingly, improvements in diagnostic accuracy and the implementation of dedicated databases and registries are expected to provide more robust epidemiologic data in the near future.

## 4. Pathogenesis of GPP

GPP is currently acknowledged as a distinct pustular neutrophilic autoinflammatory disease in which dysregulation of IL-36 cytokines plays a central and relevant pathogenic role [6,32].

IL-36 cytokines (i.e., IL-36α, IL-36β, and IL-36γ) belong to the IL-1 family, and they are variably expressed in human tissues and exert downstream effects on a number of different cell types, from keratinocytes—which are the major source of IL-36 in the skin—to epithelial cells, lymphocytes and other immune system cells.

Under physiological conditions, IL-36α and IL-36β can be found in healthy skin, while IL-36γ expression is increased in psoriasis. The production and release of IL-36γ are activated by well-characterized pathogenic cytokines classically involved in psoriasis, such as TNF-α, IL-17, and IL-22.

Importantly, IL-36 cytokines can further enhance IL-36 gene expression in keratinocytes. This leads to a positive feedback loop and a self-amplifying inflammatory process, which contributes to the progression of the disease.

It is believed that the uncontrolled expression and activation of IL-36 lead to a severe homeostatic imbalance and to a self-maintaining inflammatory cascade *via* IL-36R signaling. This disruption can be driven either by over-expression of IL-36 receptor agonists (i.e., IL-36α, IL-36β, and IL-36γ) or by a dysfunctional antagonist (IL-36RA) of the IL-36 receptor (IL-36R) [33,34,35,36].

Activation of each IL-36 isoform is mostly mediated by different proteolytic enzymes, resulting in a different degree of activation: (i) roughly a 300-fold increase in activity of IL-36α when it is activated by cathepsin G and proteinase-3; (ii) a 100- to 150-fold activation for IL-36β via cathepsin G and elastase; (iii) around a 500-fold increase for IL-36γ through neutrophil elastase. This strong enzymatic specificity explains the predominant expression of the IL-36γ isoform in the skin and airway mucosa, which are enriched in neutrophils.

As a direct result of the hyperactivated inflammatory cascade, chemokines such as CXCL1 and CXCL8 (IL-8) drive a high number of neutrophils into the epidermis. The massive accumulation of these cells results in the formation of the typical “lakes of pus” that correspond to the so-called sub-corneal “spongiform pustules of Kogoj” [37,38,39].

Genetic variants of IL-36RA (*IL36RN*) and other genes involved in the biological functions of IL-36, keratinocytes and neutrophils, such as *MPO*, *CARD15*, *SERPINA3*, *AP1S3* and *TNIP1*, have been documented in GPP patients. The identification of these genetic variations strongly confirms the relevant role of innate immunity, of IL-36 and of other inflammatory pathways in the pathogenesis of the disease [34,40,41].

Thus, the overall pathogenic profile of GPP significantly differs from that of plaque psoriasis, and therefore GPP should be considered a different immune-pathological entity. However, a crosstalk between innate and acquired immunity, occurring on a predisposing background that involves IL-17 and, somehow, IL-23, might explain the disease spectrum. This overlapping network provides a rationale for the clinical response to biologic agents approved for plaque psoriasis, as well as for the coexistence of GPP and plaque psoriasis in a subset of patients [42,43,44,45].

## 5. Clinical Features of GPP

According to the most updated international classifications and the current position of the literature, GPP has to be considered as a neutrophilic skin disease, due to the major pathogenic role played by neutrophils. The generalized form includes generalized von Zumbusch disease (i.e., the classic GPP), impetigo herpetiformis (which represents GPP occurring specifically in pregnancy), annular pustular psoriasis and paradoxical biologic-induced forms. On the other hand, localized pustular psoriasis includes palmoplantar pustular psoriasis and paradoxical forms and acrodermatitis continua of Hallopeau [2,3,46].

**Figure 1:**
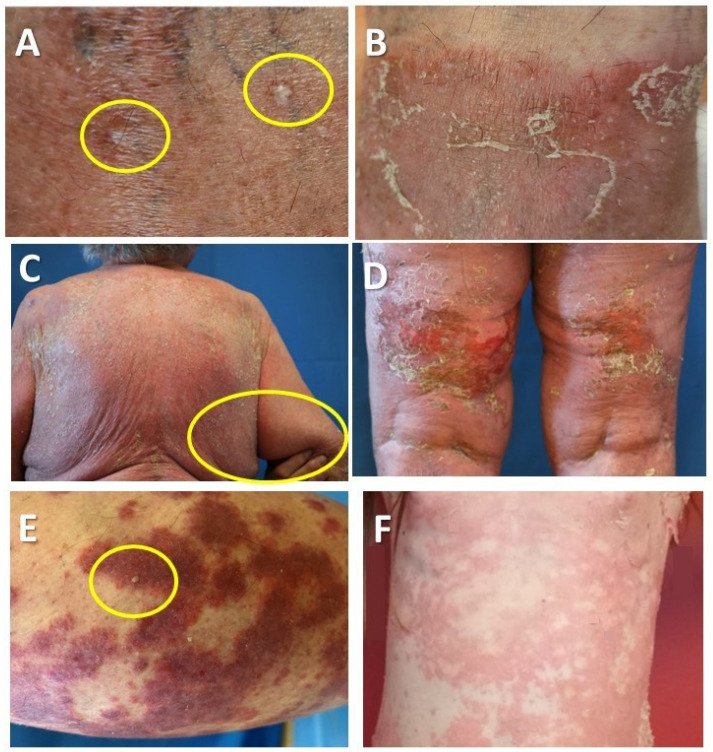
Clinical features of GPP: (**A**) White-yellowish, sterile, non-confluent pustules (yellow circle) on an erythematous background. (**B**) Coalescence of pustules with the formation of lakes of pus. This appearance is often lost in flexural areas, where friction promotes pustule rupture, resulting in large areas of superficial de-epithelialization and fine desquamation. (**C**) Diffuse erythema associated with fine desquamation and coalescent sterile pustules. The patient had severe impairment of the general condition and required assistance to stand up (yellow circle). (**D**) Superinfection of areas with ruptured lakes of pus, associated with malodorous discharge and pain. (**E**) Severe erythema with only a single residual pustule (yellow circle) after topical corticosteroid application, due to intense stinging and burning pain. The patient was unable to wear clothes or lie under bed linen. (**F**) Severe GPP with extensive skin flaking in a pediatric patient.

GPP pathognomonic lesions are characterized by the presence of primary, sterile pustules of different sizes developing on highly erythematous skin, affecting non-acral areas that can merge to form larger areas of “lakes of pus” (Figure 1).

A great effort has been made over the past years to provide simple, reproducible and applicable diagnostic criteria. In Europe the international consensus by the European Rare and Severe Psoriasis Expert Network (ERASPEN) defined GPP as the occurrence of more than one episode (relapsing) of or persistent (>3 months) primary, sterile, visible pustules on non-acral skin. Crucially, cases in which pustulation is restricted to pre-existing psoriatic plaques have to be excluded. Clinicians should also consider that GPP may present with or without systemic inflammation and with or without a history or concomitant psoriasis vulgaris. Since some cases of GPP may be difficult to diagnose clinically, histopathology could be required to confirm the clinical suspicion. This biopsy approach aligns with Japanese guidelines in which histopathology is considered mandatory for a definitive diagnosis [46,47,48,49].

GPP can occur at any age; however, it is more common in middle-aged patients (around 50 years old) [13,22,28,48,50]. An early onset of GPP could be more often observed in patients harboring the *IL36RN* mutation [26,51,52]. In terms of gender distribution, GPP can occur in both males and females; however, it seems to be more common in females [2,13,27,28,29,50].

GPP can have persistent or relapsing manifestations, with unpredictable frequency and duration of flares. Moreover, patients can exhibit residual disease between recurrences, with a variable grade of severity and cutaneous involvement [1,12,51]. It has been estimated that from 30% to 80% of patients with GPP may experience from 1 to 3 flares per year. These episodes may occur in close succession or, conversely, after longer periods of wellbeing [4,53,54,55].

Skin pain, itching and burning, a distressing needle-like sensation, chills, and edema due to subcutaneous tissue imbibition are the most commonly reported cutaneous symptoms in GPP patients [54,56].

Systemic symptoms or a tendency toward other signs or symptoms of general inflammation are not expected in all patients, reflecting a wide clinical heterogeneity. Fever can be observed in 24% to 96% of patients. Fatigue, malaise and profound asthenia are other common symptoms described in roughly half of the cases reported in the literature. Indeed, during an acute flare, patients can experience myalgia, shortness of breath, nausea, and arthralgias, as a consequence of the systemic inflammation [54,55,57,58].

## 6. GPP Triggering Factors

The sudden new onset of GPP lesions, or the exacerbation of severe flares could follow a variety of environmental and patient-specific triggers including stress, viral or bacterial infections, pregnancy, the systemic use of common medications including antibiotics and antifungal agents, and abrupt exposure to or withdrawal of corticosteroids, as well as hypocalcemia (which is often iatrogenic and frequently linked to thyroid and parathyroid diseases) [2,3,26,29,50,58].

GPP manifestations during pregnancy mostly occur during the third trimester and could be linked to hypocalcemia, other pregnancy-related endocrine and hormonal abnormalities, diminished intestinal vitamin D absorption, and emotional stress [59,60].

Some authors have also investigated the potential seasonality of acute flares of GPP [30,56]; however, to date, no significant or conclusive data have been found to support this correlation.

## 7. GPP Comorbidities

The clinical description of concomitant systemic diseases, observed and documented in GPP patients, derives mainly from hospitalization records or retrospective data collection, and, consequently, has not been fully characterized. The most common comorbidities consistently reported in the literature are essential arterial hypertension, psoriatic arthritis (PsA) and arthralgias, type 2 diabetes mellitus, metabolic liver abnormalities, and hyperlipidemia, cardiovascular disease, obesity, renal abnormalities and depression [1,26,27,50,61]. As GPP patients can frequently have concomitant chronic plaque psoriasis, these epidemiological findings are not surprising; however, recently Löfvendahl et al. [27] showed that GPP patients have an increased risk of developing or displaying some of these comorbidities compared not only with the general healthy population but also with patients with psoriasis. GPP patients can also have geographic tongue: this manifestation seems to be more frequent in patients with a genetic predisposition to GPP [26,62].

The specific association between GPP and PsA is still controversial. Diffuse arthralgia, subcutaneous tissue imbibition with associated joint movement limitation and fully diagnosed PsA have been variously reported across different studies, with ranges extending from one-fifth to one-third of GPP patients. Recently, Gershater B et al. [63] analyzed the risk of PsA in different psoriasis phenotypes, and showed that patients with plaque psoriasis had an increased risk of PsA compared to GPP patients (HR 2.3, CI 1.7–3.2, *p* < 0.0001). However, the presence of PsA and concomitant treatment for PsA should always be accurately investigated by clinicians, also considering that PsA patients with acute or uncontrolled joint symptoms could undergo inappropriate systemic corticosteroid therapy which could expose patients to GPP flares. Furthermore, a pathogenic imbalance in the IL-36 cascade has been reported in PsA and enthesitis-related juvenile idiopathic arthritis patients, suggesting a potential molecular link between these conditions [64,65].

## 8. Differential Diagnosis of GPP

**Figure 2:**
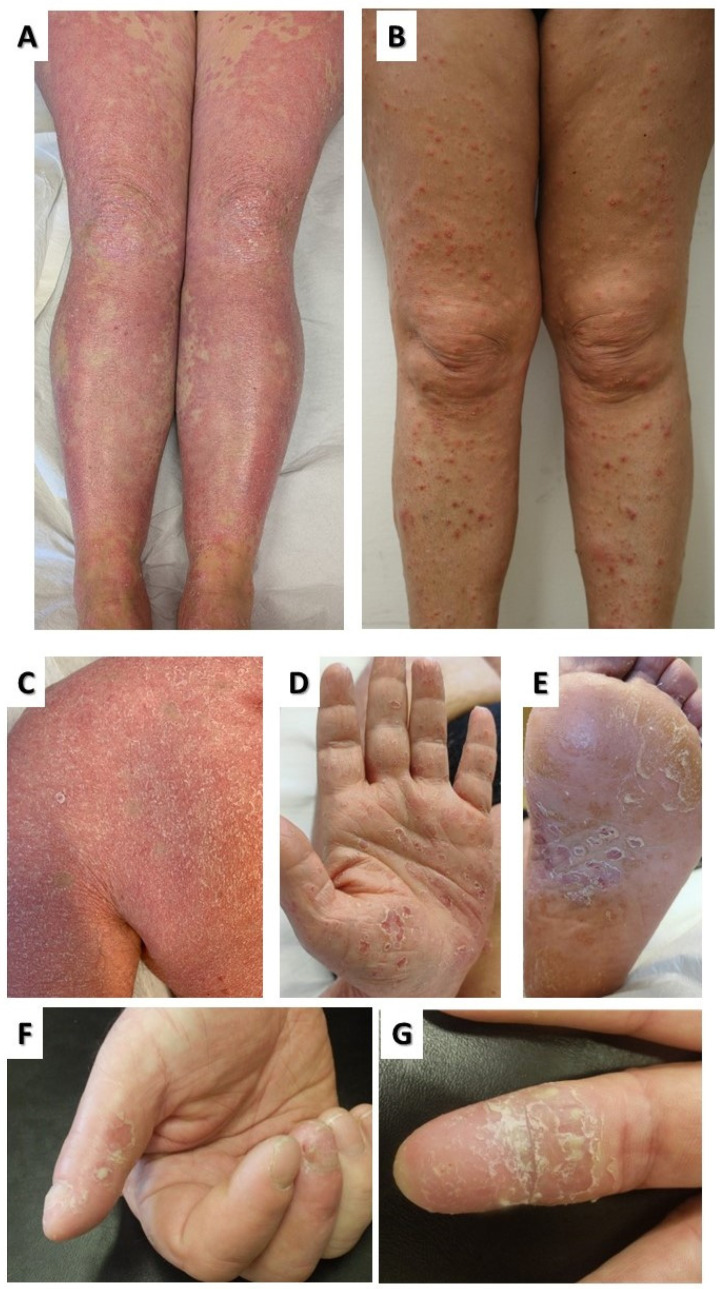
Main clinical GPP differential diagnoses: (**A**) Severe chronic plaque psoriasis. As in GPP, erythema can range from mild to severe; however, it is associated with thick, whitish scales covering the erythematous background. Upon scratching, the wax spot sign is observed, with fragmentation of the scales. No pustules are present. Systemic symptoms are usually absent, while itching may be present. (**B**) Small, sterile pustules diffusely distributed on an erythematous background. The absence of a personal or family history of psoriasis, together with a history of azathioprine intake within the previous 24 h, supported the diagnosis of AGEP, which was confirmed histopathologically. (**C**) Erythrodermic psoriasis. Diffuse erythroderma associated with fine scaling in a patient with a personal history of chronic plaque psoriasis. The absence of pustules, together with histopathological findings, was essential to establish the differential diagnosis. (**D**,**E**) Palmoplantar pustular psoriasis. Sterile pustules confined to the palms and soles. Pustules may coalesce and rupture, leaving painful erythematous and scaling areas. Localization to acral sites is an exclusion criterion for the diagnosis of GPP, as palmoplantar pustular psoriasis is considered a distinct clinical entity. (**F**,**G**) Acrodermatitis continua of Hallopeau is characterized by sterile pustules that can coalesce on the distal extremities of the fingers and toes. It may be associated with pain, functional impairment, and arthralgia, particularly when inflammation leads to nail dystrophy and osteolysis. It is considered a localized form of pustular psoriasis.

Severe chronic plaque psoriasis, localized forms of pustular psoriasis, Acute Generalized Exanthematous Pustulosis (AGEP), sub-corneal pustular dermatosis and pustular drug eruption are the main differential diagnoses of GPP (Figure 2).

The main clinical differential diagnosis of GPP is AGEP. A recent history of drug ingestion (frequently antibiotics belonging to the β-lactam class), followed by a very rapid onset (often within a short timeframe of 48 h) of small-sized sterile pustules, often localized to flexural and facial areas, with subsequent involvement of the trunk and arms, associated with erythema, edema, fever and leukocytosis, which tend to heal without recurrence within 1–2 weeks can guide clinicians toward a diagnosis of AGEP. The absence of a concurrent or personal history of plaque psoriasis and of extensive coalescence of pustules into lake of pus, together with the presence of mucosal involvement and, at histopathological examination, of eosinophils, necrotic keratinocytes in pustules, a mixed interstitial and mid-dermal perivascular infiltrate and the absence of tortuous or dilated blood vessels, are other critical criteria that can help differentiate AGEP from GPP [3,66,67].

## 9. Laboratory Tests in GPP

**Figure 3:**
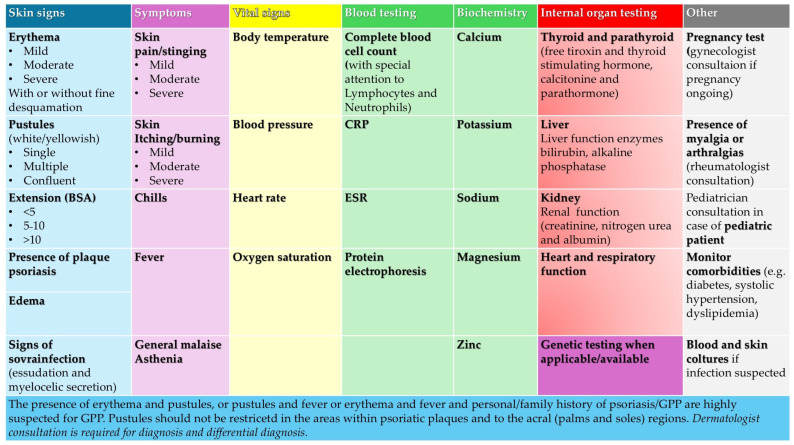
Summary of the key clinical signs, symptoms, and diagnostic workup for the diagnosis and management of an acute GPP flare (body surface area, BSA*; C-reactive protein, CRP; erythrocyte sedimentation rate, ESR. * By convention, 1% of the body surface area (BSA) is estimated to correspond approximately to the surface area of one adult hand).

No specific diagnostic blood test exists for confirming GPP; however, it is extremely important to investigate and monitor possible severe complications (internal organ dysfunction, secondary bacterial infections, systemic inflammation, dehydration, loss of plasma proteins and oligemia) (Figure 3) [48,55,68,69].

A comprehensive baseline screening should always include a complete blood cell count (with special regard to fluctuations in lymphocyte counts and the presence of marked neutrophilia), systemic inflammatory markers (e.g., ESR and CRP), blood chemistry (including urea, calcium and electrolytes), serum protein electrophoresis (showing hypoproteinemia and an altered albumin/globulin ratio), kidney and liver function tests and routine urine analysis (which may reveal albuminuria). When secondary infections are suspected, bacterial cultures form pustules fluids and blood samples may also be needed.

## 10. GPP Prognosis

The unpredictable clinical course of GPP could be profoundly different from patient to patient, but also from flare to flare within the same patient. An older age of onset, the coexistence of concomitant comorbidities and the occurrence of acute flares during pregnancy have been reported as negative prognostic factors [70].

The estimated percentage of patients requiring hospitalization [31,50,58,71,72,73] ranges up to 70%, with a calculated median duration of hospitalization of roughly one week that increases patient mortality. Hanna et al. [71] showed that the suspected diagnosis of GPP at admission, either for hospitalized patients or for patients presenting to the emergency department, was lower than at discharge, suggesting that the early inpatient management of hospitalization was also affected by limited diagnostic accuracy.

Death has been reported at a rate of up to 3.3 deaths per 100 patient-years, usually occurring as a direct consequence of septic shock and cardiac failure [26,50,72]. Most common causes of hospitalization are sepsis or unspecified bacterial infection (5–10% of patients), hepatitis, renal failure, acute respiratory distress syndrome, heart failure, as well as the progressive worsening of baseline comorbidities such as essential hypertension or gastrointestinal malabsorption or reflux or severe complications linked to treatments [21,50,73,74,75,76].

## 11. GPP Treatment

Current therapeutic guidelines for GPP are under continuous update due to the limited data, which are linked either to the low frequency of the disease or to the scarce therapeutic evidence available for anti-psoriatic conventional and biologic therapies. However, the recent advent of spesolimab has dramatically changed the overall therapeutic approach to managing GPP. Spesolimab is a selective humanized anti-IL36R biologic agent specifically approved to control acute flares of GPP. During investigational clinical studies, spesolimab demonstrated the ability to rapidly control GPP flares and to prevent disease recurrences [8,77,78,79,80,81,82,83]. Spesolimab is approved by the EMA and FDA at the dosage of 900 mg administered intravenously on day 0, in patients aged over 12 years [84,85]. A second infusion 1 week after day 0 could be administered if optimal control of cutaneous and systemic symptoms has not been reached. Delayed recurrence of flares has been demonstrated in patients treated with spesolimab *versus* those in the placebo arm.

**Figure 4:**
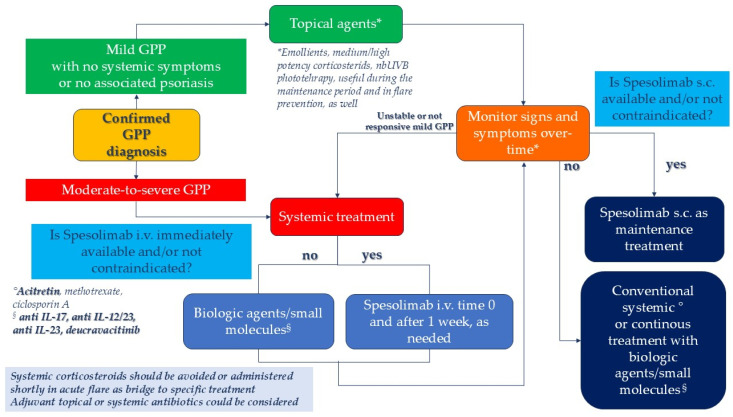
Dermatologic therapeutic approach to GPP. The choice between topical and systemic treatment is based on disease severity. Mild GPP, in the absence of systemic involvement or impairment of the patient’s general condition, may be managed with topical therapies or narrowband UVB phototherapy. However, in patients with unstable disease or an inadequate response to topical treatment, a wait-and-see approach before initiating systemic therapy is not recommended. Mild GPP is defined according to disease-specific severity scores, including the Generalized Pustular Psoriasis Area and Severity Index (GPPASI) [1] and the Generalized Pustular Psoriasis Physician Global Assessment (GPPGA) [2]. Moderate-to-severe GPP, as well as mild GPP with an unstable course or inadequate response to topical therapy, requires systemic treatment. As spesolimab is currently the only approved treatment for GPP, it should be considered the reference therapy whenever available and not contraindicated. However, in cases of limited availability, pediatric patients, pregnancy, or contraindications to spesolimab, alternative systemic therapies may be considered according to disease severity, the urgency of controlling signs and symptoms, and the patient’s history of psoriasis and/or psoriatic arthritis (PsA). During an acute flare, a short course of systemic corticosteroids may be used as bridging therapy until spesolimab, another biologic agent, or a conventional systemic therapy becomes effective. The selection of off-label treatments is based on evidence from systematic reviews and the available literature. Until additional approved therapies become available, treatment decisions will continue to rely on the available evidence, clinical judgment, and local guidelines. A proactive strategy to prevent disease flares is recommended and, whenever available and not contraindicated, should include maintenance treatment with subcutaneous spesolimab [106,107].

Moreover, spesolimab could be strategically used to prevent flares when administered subcutaneously every four weeks both in adults and adolescents. Other treatments, including anti-IL-36 pathway agents (such as imsidolimab and recibokibart) and small molecules (including deucravacitinib), are under investigation [9,86,87,88]. Before spesolimab approval, and in patients with concomitant plaque psoriasis, many other therapeutic strategies were used, and other biologic agents such as anti-IL-12/23, anti-IL-17 and anti-IL-23 agents have been associated with favorable GPP outcomes, also in clinical studies [43,89,90,91,92,93,94]. Traditional agents, with special regard to acitretin, have proven their efficacy as monotherapy or in combination with biologics, also in the maintenance period [95,96,97]. Indeed, since no predictive biomarkers for GPP flares exist, a proactive approach should be encouraged to reduce flare frequency; approved therapies for plaque psoriasis from topical treatments to phototherapy, conventional, biologics or small-molecule therapies can be used (Figure 4). Treatment of GPP during pregnancy [60,98,99,100,101] and in pediatric patients, especially those younger than 12 years of age [102,103,104,105], has to be carefully evaluated; however, there is a growing body of evidence in the literature providing insights for clinicians.

GPP treatment requires management of triggering factors and GPP complications (including infections and sepsis, dehydration, oligemia and loss of proteins, cardiac, hepatic, respiratory, renal failure) ideally in a multidisciplinary setting [1,20,43,76,96,108].

Healthcare providers, from clinicians to hospital pharmacy to payers, should consider resource availability due to the high cost of global GPP management and due to the need for prompt availability of specific drugs, including spesolimab, and supportive care units.

## 12. Clinical Practice Recommendations on GPP

**Figure 5:**
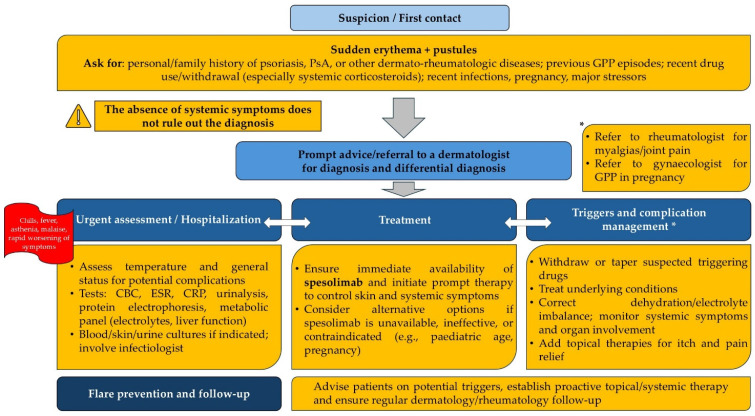
Practical algorithm for diagnosis, treatment of flares and long-term management of GPP developed during the multidisciplinary meetings. * is referred to triggers and complication management.

Clinical practice recommendations were developed as a result of the two meetings (Figure 5). The diagnosis of GPP should always be suspected, in accordance with all national and international consensus [1,3,20,46], in the presence of cutaneous manifestations such as pustules associated with erythema or fever, or in the presence of erythema and fever in patients with a personal or family history of psoriasis, PsA or GPP.

A thorough medical history should always be obtained, including the patient’s personal medical and medication history, recent infections, triggering factors, and pregnancy status, when applicable. The physician who first evaluates the patient should assess both cutaneous and systemic manifestations and carefully evaluate the patient’s overall clinical condition. Prompt consultation with a dermatologist is mandatory to confirm the diagnosis and perform the differential diagnosis. Moreover, based on the patient’s clinical signs and symptoms, laboratory investigations, assessment of vital signs, and evaluation of internal organ involvement are required.

Consultation with other specialists, such as a rheumatologist, infectious disease specialist, gynecologist, or pediatrician, may also be appropriate depending on the patient’s clinical condition and age. The decision regarding hospitalization, day-hospital management, or direct referral to a dermatologist should also be based on the patient’s overall clinical status. The management of comorbidities and systemic complications, ranging from dehydration and electrolyte imbalance to organ dysfunction, should follow local, national, and international guidelines. Specific treatment for GPP should be determined by the dermatologist.

## 13. Future Directions in GPP Management and Conclusions

GPP is a severe and potentially life-threatening disease that is often underdiagnosed and undertreated in medical practice. The distinction from psoriasis is not simply a matter of nosology but it is required to understand the unique disease genetics, immunopathogenesis, evolution and prognosis with direct therapeutic implications. Conventional systemic treatments, small molecules and biologics targeting TNF-α, IL-17, and IL-23 pathways are useful in patients with concomitant plaque psoriasis, in the maintenance therapy of GPP, or when specific therapies are not available. Indeed, biologics targeting the pathogenic IL-36 pathway provide rapid and effective control of GPP, especially in the acute phase. Future strategies to optimize GPP management must bridge critical epidemiological and clinical gaps by establishing national patient registries. These databases are essential to capture robust data and map the natural history of this rare autoinflammatory disease. Implementing standardized diagnostic criteria and identifying specific hematological biomarkers could ensure early flare recognition and could prevent misclassifications in emergency and inpatient settings.

Routine genetic screening should be included in the diagnostic workflow to guide more precise diagnosis and therapeutic choices.

As for chronic plaque psoriasis, a “bench-to-bedside approach” should lead future research and drug development.

Therefore, novel insights into etiopathogenesis and emerging treatments will offer new valuable tools for disease management; however, GPP remains a challenging and critical disease due to the lack of diagnostic and predictive biomarkers of clinical progression, treatment response, risk of flares and disease evolution. For these reasons, a well-coordinated and trained multidisciplinary approach could lead to prompt diagnosis, adequate therapies, prevention of complications and flares, standardization of care and optimization of healthcare resources.

## Data Availability

No new data were generated.
